# Can incoming United States pediatric interns be entrusted with the essential communication skills of informed consent?

**DOI:** 10.3352/jeehp.2020.17.18

**Published:** 2020-06-29

**Authors:** Nicholas Sevey, Michelle Barratt, Emma Omoruyi

**Affiliations:** Department of Pediatrics, McGovern Medical School, University of Texas Health Science Center at Houston, Houston, TX, USA; Hallym University, Korea

**Keywords:** Communication, Informed consent, Internship and residency, Spinal puncture, United States

## Abstract

**Purpose:**

According to the entrustable professional activities (EPA) for entering residency by the Association of American Medical Colleges, incoming residents are expected to independently obtain informed consent for procedures they are likely to perform. This requires residents to not only inform their patients but to ensure comprehension of that information. We assessed the communication skills demonstrated by 372 incoming pediatric interns between 2007 and 2018 at the University of Texas Health Science Center at Houston, obtaining informed consent for a lumbar puncture.

**Methods:**

During a simulated case in which interns were tasked with obtaining informed consent for a lumbar puncture, a standardized patient evaluated interns by rating 7 communication-based survey items using 5-point Likert scale from “poor” to “excellent.” We then converted the scale to a numerical system and calculated intern proficiency scores (sum of ratings for each resident) and average item performance (average item rating across all interns).

**Results:**

Interns received an average rating of 21.6 per 28 maximum score, of which 227 interns (61.0%) achieved proficiency by scoring 21 or better. Notable differences were observed when comparing groups before and after EPA implementation (76.97% vs. 47.0% proficient, respectively). Item-level analysis showed that interns struggled most to conduct the encounter in a warm and friendly manner and encourage patients to ask questions (average ratings of 2.97/4 and 2.98/4, respectively). Interns excelled at treating the patient with respect and actively listening to questions (average ratings of 3.16, each). Both average intern proficiency scores and each average item ratings were significantly lower following EPA implementation (P<0.001).

**Conclusion:**

Interns demonstrated moderate proficiency in communicating informed consent, though clear opportunities for improvement exist such as demonstrating warmth and encouraging questions.

## Introduction

### Background/rationale

In 2013, the Association of American Medical Colleges created a panel tasked with defining an integrated list of skills expected of all medical school graduates. Titled “core entrustable professional activities for entering residency,” the panel outlined 13 skills and responsibilities that an incoming intern should be capable of performing unassisted upon entering residency. Specifically, entrustable professional activity (EPA) 11 states that incoming interns are expected to independently obtain informed consent for procedures they are likely to perform [1]. Essential communication skills listed in EPA 11 and thus expected of an entrustable learner include the ability to “avoid medical jargon,” “use bidirectional communication,” “practice shared decision making” as well as “respond to emotional cues in real time.” Embodied in health care ethics [2], informed consent requires interns to not only inform their patients, but to ensure comprehension of the information provided [3]. As such, communication skills are crucial in promoting trust, empathy, and efficiency during this shared-decision making process [4]. An intern’s ability to properly communicate informed consent conversely impacts their ability to uphold the fundamental pillar of patient autonomy [2].

We are not aware of any current studies evaluating the quality of communication skills in pediatric interns while obtaining informed consent. A similar study looked at the essential components of lumbar puncture consent but did not evaluate the communication skills of the incoming interns [5]. Additionally, a scoping review indicated a need for focused, empirical studies targeting the EPAs [6].

### Objectives

The purpose of this study was to evaluate the communication skills of incoming pediatric interns while they obtain informed consent for a lumbar puncture, a common pediatric procedure for which consent is required. This study aims to indicate how “entrustable” interns are in communicating informed consent per EPA 11 guidelines.

## Methods

### Ethics statement

The study was given exempt status by the University of Texas Health Science Center at Houston Institutional Review Board (HSC-MS-15-0481).

### Study design

This was a retrospective study of an educational intervention.

### Materials and/or subjects

Each year at our mid-sized pediatric residency program, entering first year residents (interns) complete an objective structured clinical examination (OSCE) during orientation to evaluate their clinical proficiency. The 6-station, scenario-based exam provides a common baseline for all interns entering the pediatric residency program prior to beginning their clinical rotations. We analyzed OSCE performance data for all 372 interns between 2007 and 2018 at the University of Texas Health Science Center at Houston.

The specific OSCE station we evaluated involved a standardized parent (SP) with a “child” suspected of having meningitis. The intern was tasked with obtaining informed consent from the SP to perform a lumbar puncture on the “child”, one of the most common procedures consented to in general pediatrics. The child was not present, and interns had 15 minutes to complete the process using our primary teaching hospital’s blank consent form. The form remained relatively unchanged for the years included in this study. The SP then completed a 22-item survey evaluating various components of the informed consent process, 7 of which specifically evaluated the intern’s communication skills. Items in the survey’s communication section were similar to those used by multiple published communication assessment tools provided in the EPA 11 appendix including the Communication Assessment Tool, Liverpool Communication Skills Assessment Scale, and Rochester Communication Rating Scale [3] (Table 1). After the encounter, interns received feedback from the SP on their communication skills and reviewed the informed consent documentation prior to their next OSCE station.

The SP used a 5-point Likert scale ranging from “poor” to “excellent” to rate the intern on 7 communication items (Table 1). The SP-generated ratings were numerically coded for statistical analyses as follows: poor=0, fair=1, good=2, very good=3, and excellent=4. We then used these ratings to calculate 2 primary performance measures: (1) intern proficiency score and (2) average item performance.

### Technical information

The intern proficiency score measures how each intern performed across the 7 communication items as rated by their respective SP. The proficiency score was calculated by adding the intern’s 7 numerically coded ratings (up to 4 points each) with proficiency scores potentially ranging from 0 (all poor ratings) to 28 (all excellent ratings). For example, an intern who received a “very good” rating (3 points) for all 7 items would receive a proficiency score of 21. Average intern proficiency scores were calculated for all interns, pre-EPA implementation interns, and post-EPA implementation interns.

The average item performance measures the performance of each evaluated survey item averaged across multiple interns. For example, the average item performance for “conducting the encounter in a warm and friendly manner” across all interns was calculated by averaging each intern’s rating (0–4) for that specific item. Therefore, average item performance scores could potentially range from 0 (each intern was rated “poor” for that item) to 4 (each intern was rated “excellent”).

### Statistical methods

After calculating the average intern proficiency scores and average item performance, we analyzed the data for each of these performance measures in 2 ways: (1) overall analysis (all interns 2007–2018) and (2) pre-EPA versus post-EPA implementation (2007–2012 and 2013–2018, respectively). When comparing pre- and post-EPA performance, 2-way independent t-tests were used to determine significant differences in average intern proficiency and average item performance. Resulting P-values less than 0.05 were considered statistically significant. Statistics were computed using Microsoft Excel ver. 16.36 (Microsoft Corp., Redmond, WA, USA).

## Results

**Fig. 1.**
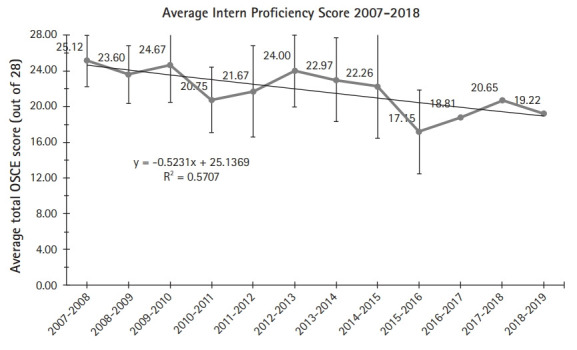
Average intern proficiency over time. OSCE, objective structured clinical examination.

Overall, intern proficiency scores ranged from 10–28 (n=372) with an average of 21.58 (Table 2). Average intern proficiency over time shows an overall slight decline with notable drops in 2010 and 2015 performance (Fig. 1). The 227 (61.0% ) interns received a score of 21 or better. However, there were notable differences when comparing the proportion of proficiency scores of at least 21 before and after EPA implementation. The 286 (76.9%) pre-EPA interns achieved proficiency scores of at least 21 compared to only 175 (47.0%) of post-EPA interns. The difference between these 2 groups was statistically significant (P<0.05), a finding that persisted across each item in the item-level analysis.

Overall, average item performance ranged from 2.97 to 3.16 across all years. Average item performance scores in the pre-EPA group ranged from 3.16 to 3.40 while the post-EPA average item performance ranged from 2.75 to 2.96. Each item’s average rating among post-EPA interns was significantly lower than those of pre-EPA interns (Table 3).

## Discussion

### Interpretation

These results reveal 2 major findings: first, there is a clear opportunity to increase the number of interns proficient at communicating informed consent, and second, efforts to improve communication should focus on demonstrating warmth and friendliness as well as encouraging questions from the patient. At first glance, it appears just over 3/5 of interns are proficient in communicating informed consent. Further scrutiny reveals a large discrepancy among proficient interns before and after EPA implementation. Nonetheless, the negative trend indicates a need for greater support and monitoring of intern communication skills during the informed consent process. Despite the large gap in proficiency between pre- and post-EPA groups, both groups shared the same 2 lowest-performing items: demonstrating warmth and friendliness and encouraging the patient’s questions. While “warmth and friendliness” is more difficult to objectively assess, studies evaluating the prevalence of what could be considered warm and friendly behavior among physicians have produced mixed results. For example, some studies found that warm and friendly behavior such as properly greeting the patient was a weakness among physicians [7] while others found this to be a strength [8]. Our findings certainly agree with studies that consistently rank the ability to encourage questions as a low-performing skill among physicians [7,9]. This particular finding reinforces a well-known issue in healthcare dubbed by some as “white-coat silence [10].” Additionally, our findings reaffirmed more directly observable strengths such as explaining terms and avoiding medical jargon [7-9].

Improvements to intern communication skills could benefit particularly with a focus on encouraging questions and potentially on encouraging social behaviors that convey warmth and friendliness (e.g., greeting the patient by name). That said, relative average item performance was similar across items within each analysis group (i.e., overall, pre-EPA, and post-EPA). Therefore, students would likely benefit from broad support during undergraduate medical education to develop these skills. Doing so would contribute to a greater proportion of incoming interns that could be entrusted to obtain and communicate informed consent as outlined by EPA 11.

There were 2 unexpectedly sharp declines during 2010 and 2015. Considering the declines as potential outliers, we found the differences between groups remained statistically significant even when each year was excluded both individually and together. While the trends observed among these 2 groupss cannot be adequately explained, they do not appear to affect our overall statistical conclusions. Intern performance on national standardized training examinations remained constant over these periods of time.

A significant drop in post-EPA performance was unexpected, particularly given that the largest drop was not observed until 2 years after EPA implementation. One possible explanation is a change in how standardized patients were trained to evaluate interns. The implementation of the EPA may have clarified expectations around the informed consent process, subsequently prompting the residency site to better align SP training with these expectations. Therefore, the observed decline may actually be the result of higher expectations by the SPs rather than a true decline in performance by the interns. Though SP information was not available for all years, available SP data for years 2009–2011 (pre-EPA) and 2017–2018 (post-EPA) revealed that one SP in particular evaluated 68 interns during that time: 46 pre-EPA interns and 22 post-EPA interns. Analysis of this SP’s ratings revealed a statistically significant drop in average intern proficiency scores (P<0.01) following EPA implementation. This finding in conjunction with the diverse medical school education of the interns evaluated by the SP supports the idea that changes to SP training/expectations may be the source of the observed differences following EPA implementation. Additional studies involving multiple sites and a greater number of SPs would likely clarify this assertion.

### Limitations

The first limitation of our study is that the data was collected from a single residency site and therefore subject to selection bias. We also did not have access to demographic information. However, interns represented over 86 medical schools across the world thereby increasing the diversity and representation in our sample, as well as external validity. A second limitation involves the subjective nature of some survey items (e.g., being warm and friendly). While SPs are trained together, it remains likely that some SPs rated interns differently due to subjective differences in how they perceived the intern during their interactions. However, the data underpinning our analyses spanned 11 years and included at least 7 SPs (based on 6 available years of SP information). Though subjective rater bias may be impossible to eliminate, standardized training combined with a variety of SPs over time limits the degree to which extreme ratings could influence the overall dataset. A third potential confounder includes the possibility of shared experiences in the same residency program contributing to similar habits developed amongst interns of the same cohort [11]. Because interns were evaluated prior to beginning their clinical rotations, influence from peers or mentors on an intern’s informed consent habits is unlikely.

### Conclusion

Overall, interns demonstrated moderate proficiency in communicating informed consent which is an EPA expected of incoming interns. Continued monitoring may clarify the trends observed in our data as well as specific areas of communication that need improvement such as growth around encouraging patients’ questions. Nonetheless, training programs (particularly undergraduate medical education) should continue to reinforce and assess communication skills in a variety of settings including during the critical informed consent process. Our data indicate a clear opportunity to increase the proportion of “entrustable learners” as they graduate medical school so that they can be expected to independently obtain informed consent from patients following EPA 11 at the start of residency.

## Figures and Tables

**Table 1.** Standardized patient survey communication items

	Items
Q1	Conducting the encounter in a warm and friendly manner.
Q2	Treating you like you are on the same level, never “talking down” to you or treating you like a child.
Q3	Letting you tell your story without interrupting.
Q4	Showing interest as a person (not acting bored).
Q5	Encouraging you to ask questions during the encounter.
Q6	Actively listening as you responded to questions.
Q7	Using easily understood words and explaining any technical or medical terms in plain language.

**Table 2.** Average intern proficiency scores (maximum score=28)

	All	Pre-EPA	Post-EPA
No. of participants	372	173	199
Average	21.58	23.28	20.1
Standard deviation	4.99	4.32	5.09
P-value		<0.001	

EPA, entrustable professional activities.

**Table 3.** Average item performance (maximum score=4)

	Q1	Q2	Q3	Q4	Q5	Q6	Q7
Overall average	2.97	3.16	3.09	3.13	2.98	3.16	3.11
Overall SD	0.87	0.79	0.86	0.81	0.88	0.81	0.82
Pre-EPA average	3.21	3.40	3.35	3.38	3.16	3.38	3.40
Pre-EPA SD	0.82	0.71	0.79	0.72	0.85	0.70	0.71
Post-EPA average	2.75	2.94	2.87	2.91	2.82	2.96	2.86
Post-EPA SD	0.86	0.79	0.87	0.82	0.87	0.84	0.83
Pre- vs. post-EPA P-values	<0.001	<0.001	<0.001	<0.001	<0.001	<0.001	<0.001

SD, standard deviation; EPA, entrustable professional activities.

## Supplementary materials

Data files are available from https://doi.org/10.7910/DVN/QDODLU

Supplement 1. Audio recording of the abstract.
